# Association between aqueous humor and vitreous fluid levels of Th17 cell-related cytokines in patients with proliferative diabetic retinopathy

**DOI:** 10.1371/journal.pone.0178230

**Published:** 2017-05-30

**Authors:** Masaru Takeuchi, Tomohito Sato, Yutaka Sakurai, Manzo Taguchi, Kozo Harimoto, Yoko Karasawa, Masataka Ito

**Affiliations:** 1 Department of Ophthalmology, National Defense Medical College, Saitama, Japan; 2 Department of Developmental Anatomy and Regenerative Biology, National Defense Medical College, Saitama, Japan; Oregon Health and Science University, UNITED STATES

## Abstract

Inflammation is known to be involved in the progression of diabetic retinopathy. We have recently reported that vitreous levels of IL-4, IL-17A, IL-22, IL-31, and TNFα are higher than the respective serum levels in proliferative diabetic retinopathy (PDR) patients, and that vitreous levels of these cytokines are higher in PDR than in other non-inflammatory vitreoretinal diseases or uveitis associated with sarcoidosis. In the present study, we investigated inflammatory cytokines including Th17 cell-related cytokines in aqueous humor samples obtained from eyes with PDR, and analyzed the association between the aqueous humor and vitreous fluid levels of individual cytokines. The study group consisted of 31 consecutive type 2 diabetic patients with PDR who underwent cataract surgery and vitrectomy for vitreous hemorrhage and/or tractional retinal detachment. Undiluted aqueous humor was collected during cataract surgery, and then vitreous fluid was obtained using a 25G vitreous cutter inserted into the mid-vitreous cavity at the beginning of vitrectomy. IL-1β, IL-4, IL-6, IL-10, IL-17A, IL-17F, IL-21, IL-22, IL-23, IL-25, IL-31, IL-33, IFN-γ, soluble CD40 ligand (sCD40L), and TNFα levels in the aqueous humor and vitreous fluid were measured using a beads-array system. Although IL-17A was detected in the aqueous humor of eyes with PDR and the level correlated with IL-17A level in the vitreous fluid, both percent detectable and level of IL-17A in the aqueous humor were significantly lower than those in the vitreous fluid. Vitreous IL-17A level was related significantly to IL-10, IL-22, and TNFα levels in aqueous humor as well as in vitreous fluid, On the other hand, aqueous IL-17A level was not related significantly to aqueous or vitreous levels of IL-10, IL-22 or TNFα level. The present study demonstrated that IL-17A level and detectable rate in the aqueous humor of patients with PDR are markedly lower than those in the vitreous fluid and aqueous IL-17A does not correlate with vitreous levels of other cytokines, and hence should not be used as a surrogate for IL-17A in the vitreous fluid.

## Introduction

Diabetic retinopathy (DR) leading to blindness is one of the most severe complications of diabetes. The prevalence of DR and vision-threatening DR in diabetic patients aged 40 years and older in the USA was 28.5% and 4.4%, respectively [1]. In a Japanese study, the incidence of DR in type 2 diabetic patients who had no DR at baseline was 26.6% over 8 years, and the risk of progression to proliferative DR (PDR) in those with mild non-proliferative DR was 15.9% [2].

Chronic inflammation under sustained hyperglycemic condition dysregulates the retinal microvasculature and provokes DR [3, 4]. Neovascularization followed by fibrovascular changes are characteristics of PDR, and result in vitreous hemorrhage or tractional retinal detachment [5]. Various angiogenic factors such as growth factors and inflammatory cytokines have been identified in eyes with PDR, and are implicated in the progression of DR [5–11].

T helper (Th) 17 cells, which preferentially produce interleukin (IL)-17 (also called IL-17A), IL-17F, IL-21, and IL-22 [12], are involved in various inflammatory and autoimmune diseases [13]. IL-17A plays a critical role in the recruitment and activation of immune cells, and induces the production of proinflammatory cytokines by non-immune cells such as fibroblasts, endothelial cells, and epithelial cells [14]. Serum level of IL-17 is elevated in patients with PDR compared with controls [15, 16], and Chernykh et al. [17] report that IL-17A level is also increased in the vitreous fluid in eyes with PDR. We recently reported that vitreous levels of IL-4, IL-17A, IL-22, IL-31, and TNFα in PDR patients were higher than the respective levels in serum, and that vitreous levels of these cytokines were higher in PDR than in idiopathic epiretinal membrane (ERM), macular hole (MH), or uveitis associated with sarcoidosis [18]. On the other hand, IL-17 is also known as an angiogenic factor. Fibroblasts and monocytes stimulated in vitro with IL-17 produce vascular endothelial growth factor (VEGF)-A [19], and IL-17 released in the tumor microenvironment renders tumor resistance to anti-VEGF therapy [20]. In addition, upregulation of VEGF via IL-17 promotes development of microvessel structures in rheumatoid arthritis and tumor growth [21, 22]. In the eye, IL-17 facilitates laser-induced choroidal neovascularization in a VEGF-independent manner [23], and involvement of IL-17 in age-related macular degeneration has been implicated [24–26]. IL-17A expression is also observed in the cornea of eyes infected with herpes simplex virus, which promotes corneal neovascularization via VEGF-A [27]. However, little is currently known about the involvement of IL-17 in the development and progression of DR.

Aqueous humor sample can be collected more easily and repeatedly compared with vitreous fluid. Several studies have shown that IL-6 and VEGF are elevated in the aqueous humor of patients with DR [28–30]. However, IL-17A level in aqueous humor and the relation with other inflammatory cytokines including IL-6 and VEGF in diabetic patients with DR or PDR remain unclear. In the present study, we investigated aqueous humor levels of IL-17A and other inflammatory cytokines, and analyzed the association between aqueous humor and vitreous fluid levels of individual cytokines.

## Subjects and methods

### Subjects

The study group consisted of 31 consecutive type 2 diabetic patients with PDR who underwent cataract surgery and vitrectomy for vitreous hemorrhage and/or tractional retinal detachment between January 1 and July 31, 2016 in National Defense Medical College. The exclusion criteria were previous vitrectomy, prior intravitreal therapies, trauma, uveitis, and infectious endophthalmitis. The age (mean ± SD) was 56.8 ± 12.4 years (range 36–84), and gender (male/female) ratio was 24/7. Retinal photocoagulation had been performed in 25 eyes (80.6%) before vitrectomy, and had not been performed in 6 eyes. Vitreous hemorrhage (VH) obscuring fundus findings was observed in 25 of 31 eyes (80.6%). In 8 eyes (25.8%) with or suspected of severe tractional retinal detachment caused by active fibrovascular membrane, intravitreal injection of 1.25 mg/0.05 mL bevacizumab (IVB) was performed 2 or 3 days before vitrectomy to minimize intraoperative hemorrhage.

### Ethics statement

The present study was approved by the Institutional Review Board of National Defense Medical College and followed the tenets of the Declaration of Helsinki. Informed consent was acquired from all participants who signed a copy of the Institutional Review Board approved consent form prior to participation.

### Sample collection

Approximately 0.2 to 0.5 mL of undiluted aqueous humor was collected during cataract surgery when the anterior chamber was replaced by viscoelastic substance. After cataract surgery, vitreous fluid was obtained using a 25G vitreous cutter inserted into the mid-vitreous cavity at the beginning of vitrectomy before active infusion. Aqueous humor and vitreous samples were transferred into sterile tubes and stored at -70°C until analysis. The vitreous samples were centrifuged to remove cellular components. No complication associated with sampling was observed.

### Cytokine measurements

Cytokines in aqueous humor and vitreous fluid samples were measured by the Bio-Plex Pro™ Human Th17 Cytokine Assays^®^ (Bio-Rad Laboratories, Inc. Tokyo, Japan) per manufacturer's instructions as described previously [18]. The following cytokines were measured: IL-1β, IL-4, IL-6, IL-10, IL-17A, IL-17F, IL-21, IL-22, IL-23, IL-25, IL-31, IL-33, IFN-γ, soluble CD40 ligand (sCD40L), and TNFα. The lower limits of detection obtained from our standard curves, as described in detail previously [18] were 0.27–0.28 pg/ml for IL-1β, 1.3–4.3 pg/ml for IL-4, 0.9–2.6 pg/ml for IL-6, 2.0–3.7 pg/ml for IL-10, 1.5–2.0 pg/ml for IL-17A, 1.8–8.0 pg/ml for IL-17F, 5.0–20.5 pg/ml for IL-21, 4.6–5.0 pg/ml for IL-22, 7.0–26.1 pg/ml for IL-23, 1.2–1.4 pg/ml for IL-25, 2.8–4.8 pg/ml for IL-31, 3.3–7.8 pg/ml for IL-33, 3.2–3.9 pg/ml for IFN-γ, 3.2–7.4 pg/ml for sCD40L, and 0.3–2.4 pg/ml for TNFα.

### Statistical analysis

Percent of samples with detectable cytokine in the aqueous humor and vitreous fluid is presented as percent detectable (%), and was compared by Pearson's chi-squared test. Comparison of cytokine levels between aqueous humor and vitreous fluid was performed using a nonparametric test (Wilcoxon signed-rank test). Cytokines with values below limits of detection were graded as negative and the levels were assigned a numerical value of 0 pg/ml for statistical analysis. Spearman’s correlation coefficient test and multiple regression analysis were used to assess the correlation between cytokine levels in aqueous humor and vitreous fluid. A *P* value less than 0.05 was considered significant.

## Results

### Comparison of cytokines in aqueous humor and vitreous fluid

**Fig 1:**
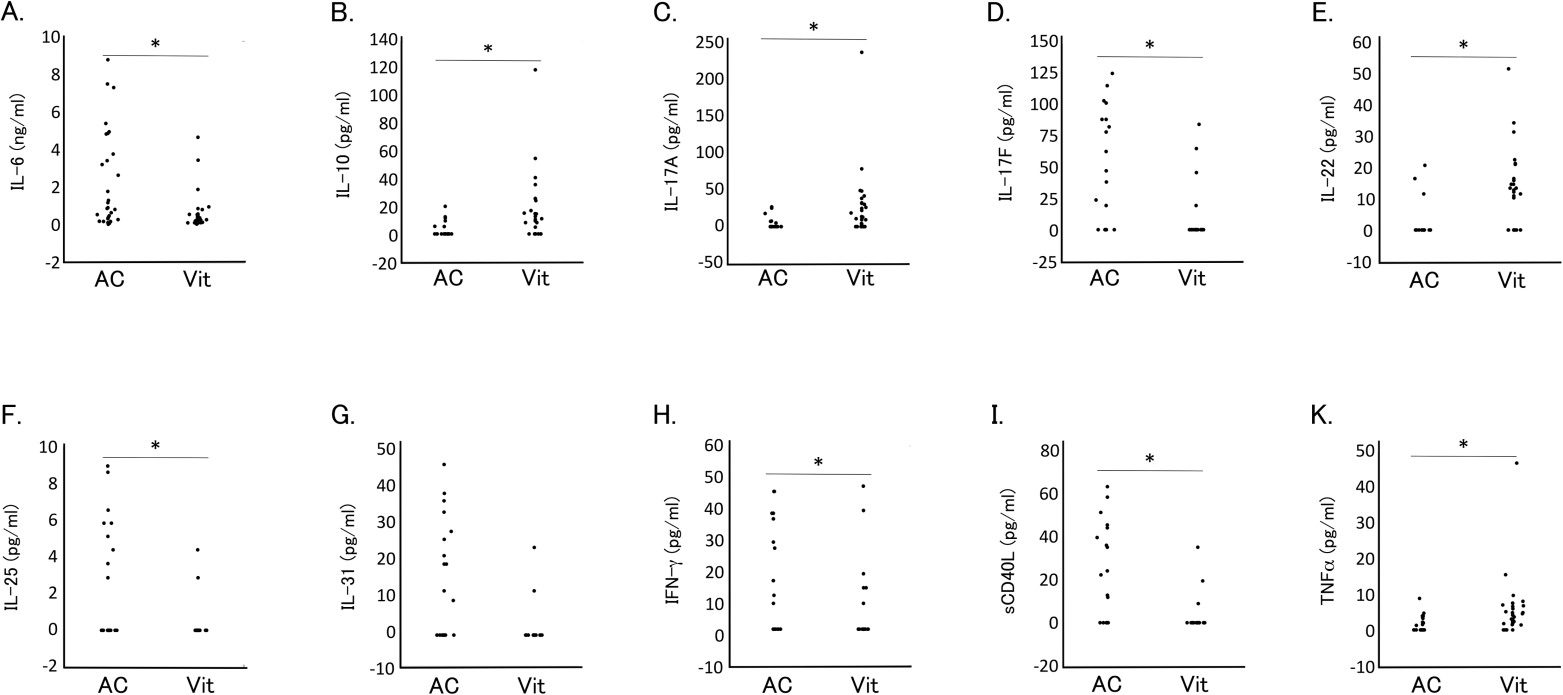
Comparison of cytokine levels in aqueous humor and vitreous fluid of patients with proliferative diabetic retinopathy. Levels of (A) IL-6, (B) IL-10, (C) IL-17A, (D) IL-17F, (E) IL-22, (F) IL-25, (G) IL-31, (H) IFN-γ, (I) sCD40L, and (J) TNFα in aqueous humor (AC) and vitreous fluid (Vit) are shown as dot plots. * *P* < 0.05 by Wilcoxon signed-rank test.

Percent detectable and levels of cytokines in the aqueous humor and vitreous fluid collected from 31 eyes with PDR are shown in Table 1. Cytokines with percent detectable higher than 30% either in the aqueous humor or in the vitreous fluids were IL-6, IL-10, IL-17A, IL-17F, IL-22, IL-25, IL-31, IFN-γ, sCD40L, and TNFα. Among these cytokines, IL-10, IL-17A, IL-22, and TNFα were detected at significantly lower percentage in the aqueous humor than in the vitreous fluid, while IL-17F, IL-25, IL-31, IFN-γ, and sCD40L were detected at significantly (except IL-31, not significant) higher percentage, and IL-6 was detected in 100% of both aqueous humor and vitreous fluid samples. Fig 1 shows the dot plots of levels of cytokines with percent detectable higher than 30% in either the aqueous humor or vitreous fluid. Similar to the percent detectable results, aqueous humor levels of IL-10, IL-17A, IL-22, and TNFα were significantly lower than the respective levels in vitreous fluid, while aqueous levels of IL-17F, IL-25, IFN-γ, and sCD40L were significantly higher than the respective vitreous levels. Aqueous IL-6 level was significantly higher than the vitreous level.

**Table 1:** Cytokines detected in aqueous humor and vitreous fluid samples collected from patients with proliferative diabetic retinopathy.

	Aqueous humor (n = 31)		Vitreous fluid (n = 31)	
	No. (%) detectable	Level (pg/ml) mean ± SD	No. (%) detectable	Level (pg/ml) mean ± SD
**IL-1β**	2 (6.5)	0.04 ± 0.15	0 (0)	0
**IL-4**	2 (6.5)	0.64 ± 2.51	3 (9.7)	0.5 ± 1.87
**IL-6**	31 (100)	2245 ± 2581^†^	31 (100)	626.7 ± 1021
**IL-10**	6 (19.4)	2.08 ± 4.85	18 (58.1)*	14.6 ± 23.8^†^
**IL-17A**	6 (19.4)	2.89 ± 7.19	21 (67.8)*	24.4 ± 43.9^†^
**IL-17F**	14 (45.2)*	33.6 ± 43.4^†^	4 (12.9)	6.85 ± 20.1
**IL-21**	6 (19.4)	13.5 ± 28.5	2 (6.5)	14.6 ± 63.1
**IL-22**	3 (9.7)	1.56 ± 4.98	18 (58.1)^*^	10.8 ± 12.3^†^
**IL-23**	0 (0)	0	0 (0)	0
**IL-25**	10 (32.3)*	1.76 ± 2.85^†^	2 (6.5)	0.23 ± 0.93
**IL-31**	12 (38.7)	9.24 ± 13.5	6 (19.4)	3.74 ± 9.41
**IL-33**	2 (6.5)	0.84 ± 3.29	0 (0)	0
**IFN-γ**	11 (35.5)*	11.2 ± 17.2^†^	2 (6.5)	1.38 ± 5.63
**sCD40L**	12 (38.7)*	14.3 ± 20.8^†^	3 (9.7)	2.04 ± 7.17
**TNFα**	11 (35.5)	1.18 ± 2.02	27 (87.1)^*^	5.58 ± 8.24^†^

No. (%) detectable: Number (%) of samples with detectable cytokine.

^*^ *P* < 0.05 aqueous humor vs. vitreous fluid, by Pearson's chi-squared test.

^†^ *P* < 0.05 aqueous humor vs. vitreous fluid, by Wilcoxon signed-rank test.

### Association of aqueous with vitreous cytokine levels

**Fig 2:**
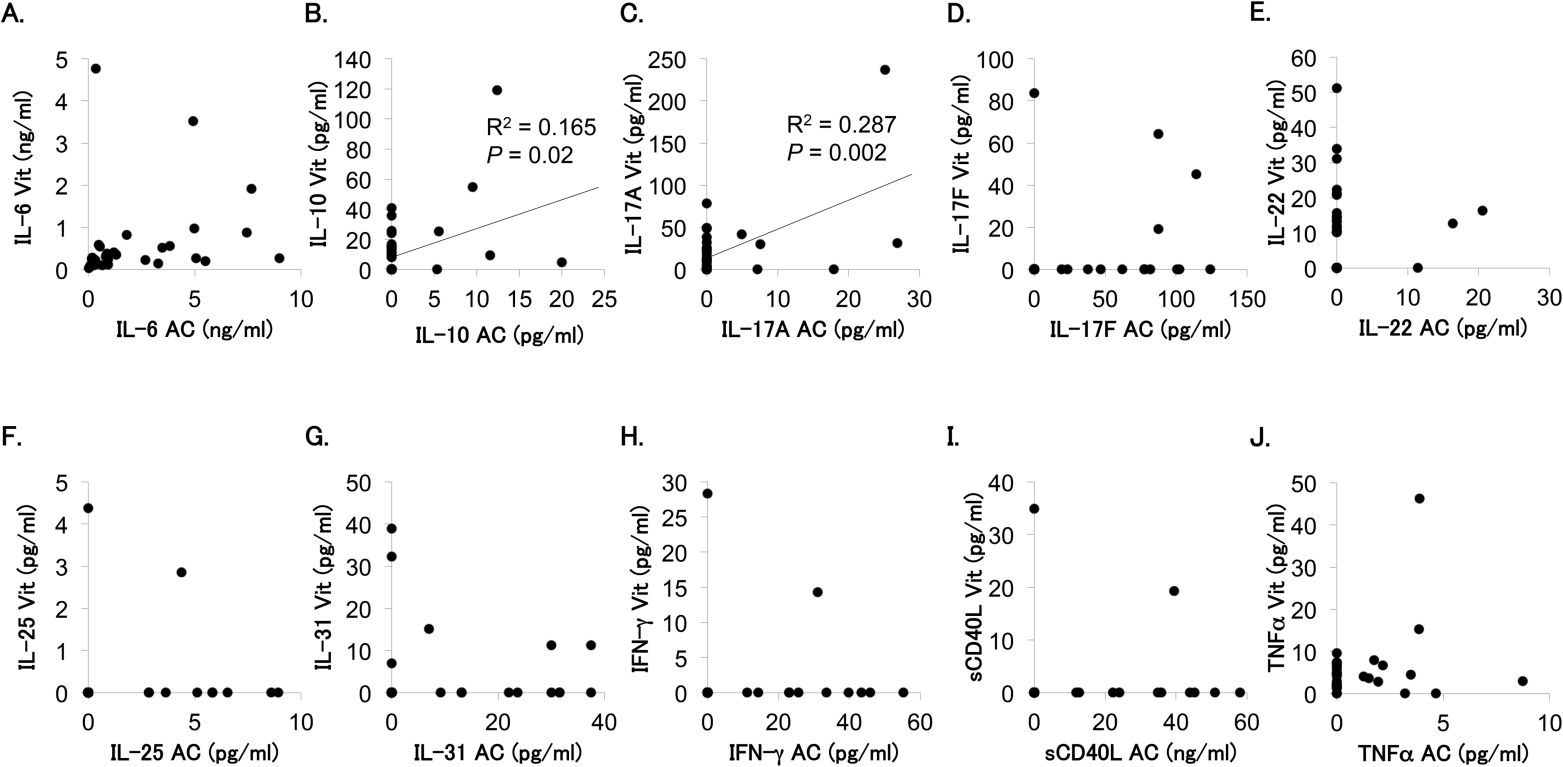
Association of cytokine levels in aqueous humor and vitreous fluid of patients with proliferative diabetic retinopathy. Correlation of aqueous level (AC, x-axis) vs. vitreous level (Vit, y-axis) for each cytokine was analyzed by Spearman’s correlation coefficient test. (A) IL-6: y = 0.45 + 0.08x, R^2^ = 0.039, *P* = 0.284; (B) IL-10: y = 10.4 + 1.99x, R^2^ = 0.165, *P* = 0.02; (C) IL-17A: y = 14.9 + 3.27x, R^2^ = 0.287, *P* = 0.002; (D) IL-17F: y = 3.66 + 0.09x, R^2^ = 0.042, *P* = 0.269; (E) IL-22: y = 10.8 + 0.03x, R^2^ = 0.0001, *P* = 0.956; (F) IL-25: y = 0.24–0.001x, R^2^ = 0.00001, *P* = 0.988; (G) IL-31: y = 4.08–0.04x, R^2^ = 0.003, *P* = 0.775; (H) IFN-γ: y = 1.41–0.004x, R^2^ = 0.0001, *P* = 0.9519; (I) sCD40L: y = 1.58 + 0.03x, R^2^ = 0.009, *P* = 0.617; and (J) TNFα: y = 4.42 + 0.99x, R^2^ = 0.059, *P* = 0.188.

Fig 2 presents the correlation between aqueous and vitreous levels of IL-6, IL-10, IL-17A, IL-17F, IL-22, IL-25, IL-31, IFN-γ, sCD40L, and TNFα. A significant correlation was observed in IL-10 and IL-17A levels. Subsequently, we examined the relationship of IL-17A level with other cytokine levels in the aqueous humor and in the vitreous fluid by multiple regression analysis (Table 2). Vitreous IL-17A level was significantly related to vitreous level of IL-6, IL-10, IL-22, or TNFα, while aqueous IL-17A level was related to aqueous level of IL-25 but not with that of IL-10, IL-22, or TNFα.

**Table 2:** Multiple regression analysis of IL-17A level with other cytokine levels in aqueous humor and vitreous fluid of patients with proliferative diabetic retinopathy (PDR).

	Variable	Estimate	95% CI	P value
IL-17A (Vit)^*^ Adjusted R^**2**^ **= 0.915** **P < 0.0001**	IL-6 (Vit)	-0.03	-0.05 ~ -0.001	0.0181
	IL-10 (Vit)	-0.80	-1.56 ~ -0.04	0.0395
	IL-17F (Vit)	1.04	-0.72 ~ 2.80	0.2335
	IL-22 (Vit)	1.14	0.60 ~ 1.67	0.0002
	IL-25 (Vit)	12.6	-46.8 ~ 72.0	0.6637
	IL-31 (Vit)	-0.66	-1.54 ~ 0.23	0.1366
	IFN-γ (Vit)	1.06	-12.0 ~ 14.1	0.8676
	sCD40L (Vit)	-0.14	-8.97 ~ 8.69	0.9733
	TNFα (Vit)	6.35	4.23 ~ 8.48	<0.0001
**IL-17A (AC)** ^**†**^ **Adjusted R**^**2**^ **= 0.333** **P = 0.0308**	IL-6 (AC)	0.0004	-0.004 ~ 0.005	0.8661
	IL-10 (AC)	-0.88	-2.02 ~ 0.26	0.1234
	IL-17F (AC)	0.02	-0.24 ~ 0.28	0.8889
	IL-22 (AC)	0.56	-0.43 ~ 1.54	0.2532
	IL-25 (AC)	-7.21	-13.4 ~ -1.03	0.0244
	IL-31 (AC)	0.99	-0.23 ~ 2.22	0.1054
	IFN-γ (AC)	0.71	-0.33 ~ 1.74	0.1690
	sCD40L (AC)	-0.32	-1.47 ~ 0.82	0.5618
	TNFα (AC)	1.79	-0.19 ~ 3.77	0.0735

CI: confidence intervals.

^*^ Cytokines in vitreous fluid

^†^ cytokines in aqueous humor.

### Relationship between vitreous IL-17A level with other cytokine levels in aqueous humor or between aqueous IL-17A level and other cytokine levels in vitreous fluid

We examined the relationship between vitreous IL-17A level and aqueous levels of various cytokines as well as between aqueous IL-17A level and vitreous cytokine levels (Tables 3 and 4) by multiple regression analysis. Vitreous IL-17A level was significantly related to aqueous IL-10, IL-22, and TNFα levels in addition to aqueous IL-17A level (Table 3). On the other hand, aqueous IL-17A level was not related to any of the cytokine levels in vitreous fluid (Table 4).

**Table 3:** Multiple regression analysis of IL-17A level in vitreous fluid with cytokine levels in aqueous humor of patients with proliferative diabetic retinopathy (PDR).

	Variable	Estimate	95% CI	P value
**IL-17A (Vit)** ^*^ Adjusted R^**2**^ **= 0.615** **P = 0.0004**	IL-6 (AC) ^†^	-0.013	-0.03 ~ 0.01	0.2171
	IL-10 (AC)	12.3	6.64 ~ 17.9	0.0002
	IL-17A (AC)	4.59	2.48 ~ 6.71	0.0002
	IL-17F (AC)	0.71	-0.51 ~ 1.93	0.2378
	IL-22 (AC)	-8.28	-13.0 ~ -3.55	0.0016
	IL-25 (AC)	14.4	-18.2 ~ 47.0	0.3677
	IL-31 (AC)	-2.08	-8.15 ~ 3.98	0.4819
	IFN-γ (AC)	0.91	-4.14 ~ 5.95	0.7117
	sCD40L (AC)	-1.64	-7.02 ~ 3.74	0.5332
	TNFα (AC)	-12.6	-22.6 ~ -2.66	0.0156

CI: confidence intervals.

^*^ Cytokines in vitreous fluid

^†^ cytokines in aqueous humor.

**Table 4:** Multiple regression analysis of IL-17A level in aqueous humor with cytokine levels in vitreous fluid of patients with proliferative diabetic retinopathy (PDR).

	Variable	Estimate	95% CI	P value
**IL-17A (AC)** ^*^ Adjusted R^**2**^ **= 0.425** **P = 0.0125**	IL-6 (Vit) ^†^	-0.01	-0.02 ~ 0.01	0.3805
	IL-10 (Vit)	0.28	-0.08 ~ 0.64	0.1214
	IL-17A (Vit)	-0.03	-0.22 ~ 0.16	0.7583
	IL-17F (Vit)	0.14	-0.63 ~ 0.92	0.7081
	IL-22 (Vit)	-0.13	-0.45 ~ 0.18	0.3892
	IL-25 (Vit)	18.7	-6.75 ~ 44.1	0.1410
	IL-31 (Vit)	0.28	-0.12 ~ 0.68	0.1553
	IFN-γ (Vit)	-1.93	-7.50 ~ 3.64	0.4782
	sCD40L (Vit)	-0.75	-4.51 ~ 3.01	0.6819
	TNFα (Vit)	0.06	-1.47 ~ 1.59	0.9361

CI: confidence intervals.

^*^ Cytokines in aqueous humor

^†^cytokines in Vitreous fluid.

### Association of IL-17F level with other cytokine levels in aqueous humor

**Fig 3:**
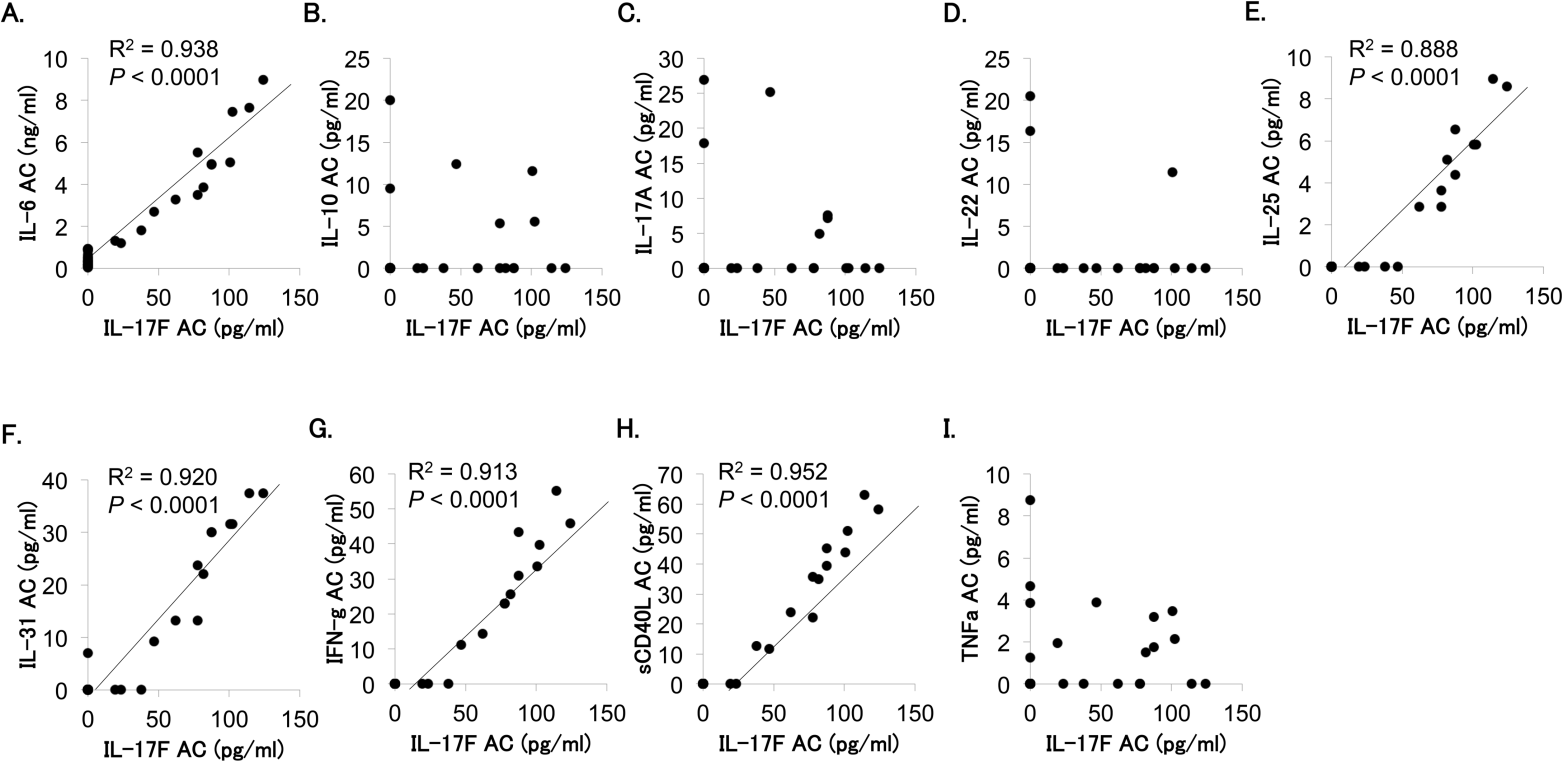
Association of IL-17F level with levels of other cytokines in aqueous humor of patients with proliferative diabetic retinopathy. Correlation of aqueous (AC) IL-17F level (x-axis) vs. aqueous levels of other cytokines (y-axis) was analyzed by Spearman’s correlation coefficient test. (A) IL-6: y = 306.1 + 57.7x, R^2^ = 0.938, *P* < 0.0001; (B) IL-10: y = 1.74 + 0.010x, R^2^ = 0.008, *P* = 0.6338; (C) IL-17A: y = 2.98–0.003x, R^2^ = 0.0002, *P* = 0.9347; (D) IL-22: y = 1.84–0.008x, R^2^ = 0.005, *P* = 0.6964; (E) IL-25: y = -0.32 + 0.06x, R^2^ = 0.888, *P* < 0.0001; (F) IL-31: y = -0.82 + 0.30x, R^2^ = 0.920, *P* < 0.0001; (G) IFN-γ: y = -1.60 + 0.38x, R^2^ = 0.913, *P* < 0.0001; (H) sCD40L: y = -1.51 + 0.47x, R^2^ = 0.952, *P* < 0.0001; and (I) TNFα: y = 1.10 + 0.002x, R^2^ = 0.002, *P* = 0.8076.

On the other hand, IL-17F was the Th17 cell-related cytokine more frequently detected in aqueous humor than in vitreous fluid. Therefore, we examined the correlation between IL-17F level and levels of other cytokines in the aqueous humor. As shown in Fig 3, aqueous IL-17F level correlated significantly with aqueous level of IL-6, IL-25, IL-31, IFN-γ and sCD40L.

## Discussion

Currently, the principal treatments for DR are strict control of blood glucose and blood pressure, and lipid-lowering therapy in addition to local ocular treatment [31, 32]. Although DR progresses mainly in patients with poor glucoregulation, gradual changes in the eye with DR are hardly reflected by systemic laboratory investigations for monitoring treatment efficacy. The progression of DR can be predicted more precisely by examining local factors. Recent accumulating evidence implies the involvement of inflammation in the pathogenesis of DR [3, 4], suggesting that identification of the correlation between inflammatory factors and DR may provide critical clues on the diagnosis, prevention, and treatment of DR. We have previously reported that Th2 and Th17 cell-related cytokines; specifically IL-4, IL-10, IL-17A, IL-22, IL-31and TNFα, are exclusively elevated in the vitreous fluid of PDR compared with that of ERM, MH, or uveitis associated with sarcoidosis [18]. However, since it is not possible to collect vitreous fluid repeatedly from the same patients with PDR, vitreous fluid is not an appropriate sample for monitoring biomarkers of PDR progression. Therefore, in the present study, we assayed inflammatory cytokines in intraocular fluids to identify those that are frequently detected (more than 30% of samples) in either the aqueous humor or vitreous fluid, and investigated in detail the correlation between aqueous and vitreous levels of these cytokines. We identified 10 cytokines that were frequently detected in the aqueous humor or/and vitreous fluid of PDR eyes, including IL-10, IL-17A, IL-31 and TNFα, which were found to be elevated in vitreous samples in our previous study [18], and a correlation was observed between aqueous and vitreous levels for IL-10 and IL-17A (Fig 2). Although these findings suggest that IL-17A in aqueous humor is the most suitable cytokine that reflects inflammatory cytokines characteristically elevated in the vitreous of PDR, the percent detectable and the mean level of IL-17A in aqueous humor were distinctly lower than those in vitreous fluid (Table 1). In addition, aqueous IL-17A level was not related to vitreous level of IL-10, IL-17A or TNFα by multiple regression analysis (Table 4), although significant correlation between aqueous IL-17A and aqueous IL-10, and TNFα levels was observed (S1 Fig). It is necessary to consider alternative cytokines or methods that allow monitoring of the progression of DR using the aqueous humor.

Our results showed that in PDR, IL-17A level in aqueous humor correlated with that in vitreous fluid, although the level and percent detection of IL-17A in aqueous humor were lower than those in vitreous fluid. A similar pattern was observed for IL-10 in primary vitreoretinal lymphoma (PVRL). In PVRL, IL-10 in the vitreous fluid is elevated and vitreal IL-10 to IL-6 ratio higher than 1.0 is considered to indicate PVRL rather than non-infectious uveitis [33]. Kuiper et al. [35] measured IL-10 level in the aqueous humor of PVRL patients and found that IL-10 in aqueous humor is much lower than that in vitreous fluid, although there was a significant correlation between aqueous and vitreous IL-10 levels as well as IL-10 to IL-6 ratio [34]. Whether this trend exists with key cytokines in other ocular diseases remains to be examined.

Since vitreous IL-17A level was related to both vitreous and aqueous levels of IL-10, IL-22, and TNFα, it is possible that immune responses mediated by these cytokines are evoked not only in the posterior segment of eyes with PDR and also in the anterior segment. On the other hand, the most frequently detected Th17-related cytokine in the aqueous humor of eyes with PDR was IL-17F, and not IL-17A (Table 1). Furthermore, aqueous IL-17F level correlated with aqueous levels of IL-6, IL-25, IL-31, IFN-γ, and sCD40L, while aqueous IL-17A was not related to aqueous or vitreous level of most cytokines examined (Table 2). Both IL-17A and IL-17F are hallmark cytokines of Th17cells, but they are also produced by a wide variety of innate immune cells including γδT cells, several subtypes of type 3 innate lymphoid cells (ILC3s), natural killer (NK) cells, NKT cells and neutrophils [35]. IL-17F shares approximately 50% amino acid sequence homology with IL-17A, and both upregulate the expression of proinflammatory cytokines and chemokines via NF-κB, MAPK and C/EBP activation by binding to IL-17 receptor A (IL-17 RA) and IL-17 RC complex on target cells [36]. Although IL-17A and IL-17F are involved in the development of inflammation in a similar manner, IL-17A binds to IL-17RA with higher affinity while IL-17F has higher affinity to IL-17RC [37]. In addition, the distributions of IL-17RA and IL-17RC in tissues and cells differ; IL-17RA is expressed mainly on immune cells, while IL-17RC is preferentially expressed on nonimmune cells [38]. Since IL-17F is a weaker inducer of proinflammatory cytokines and is produced by a wider range of cell types including epithelial cells compared with IL-17A [36], it is likely that IL-17F independently evokes similar inflammation in the anterior segment of the eye with PDR via distinct immune pathway from that of IL-17A. We are currently undertaking a study to examine whether elevation of IL-17F in the aqueous humor is specific to PDR by comparing with other ocular diseases.

The pathological roles of IL-17 in the development of DR remain unclear. However, previous study has shown that serum level of IL-17 was elevated in type 2 diabetes mellitus patients with poor glucoregulation compared to those with good glucoregulation, and that reduction of HbA1c upon treatment was associated with a significant decrease in serum IL-17 level [39]. In addition, Xu et al. [40] have demonstrated that the ratio of IL-17A^+^CD4^+^ T cells in peripheral blood mononuclear cells increased in rats with streptozotocin (STZ)-induced type 1 diabetes, and intravitreal injection of anti-IL 23Rp19 antibody, which blocks the IL-23-Th17-IL-17A pathway, improved the blood-retinal barrier function in STZ-treated rats.

Vitreous levels of most cytokines, including IL-17A, in PDR patients were lower in the present study compared with our previous study [18]. A possible explanation for this discrepancy is the difference in sample preparation procedure between the two studies. In the previous study, vitreous samples were not centrifuged to remove cellular and other components, and collected samples were transferred into sterile tubes and stored at -70°C until analysis. In the present study, vitreous samples were centrifuged after collection, and the supernatants were frozen. Therefore, cytokines present in the vitreous gel or in cells were removed in the present study. The differences between the two studies raise an important point of the need to standardize sample collection, preparation and test methods when comparing results between centers or studies.

In a previous study, we compared the vitreous levels of cytokines between PDR eyes with and without VH, and reported no significant differences between PDR patients with and those without VH both in percent detectable and mean vitreous levels of all the cytokines tested in that study [18]. In the present study, VH was not observed in the patient with the highest vitreous levels of IL-10, IL-17A and TNFα. However, since PDR is commonly associated with VH, it is difficult to remove the bias of VH in a study of consecutive PDR eyes.

Some studies have indicated that vitreous IL-10 level in DR patients is significantly higher than that in patients with ERM or MH [41, 42]. In our previous study also, IL-10 was detected in the vitreous of PDR patients, but not in the vitreous of patients with ERM or MH [18]. The present study demonstrated a significant correlation between aqueous IL-10 level and vitreous IL-10 level. Aqueous level of VEGF has been shown to correlate positively with macular thickness and severity of macular edema, whereas aqueous IL-10 level correlates inversely with severity of macular edema [43]. In the present study also, IL-10 was negatively related to IL-17A in vitreous fluid (Table 2), it is possible that vitreous IL-10 may play an inhibitory role on the development of PDR to counteract the effect of IL-17A.

## Conclusions

The present study demonstrated that in patients with PDR, IL-17A was detected in the aqueous humor, and correlated significantly with vitreous IL-17A. However, the percent detectable and mean level of IL-17A in aqueous humor were significantly lower than those in vitreous fluid, and a relationship between IL-17A level and IL-10, IL-22, and TNFα levels was observed in vitreous fluid but not in aqueous humor. Therefore, aqueous humor IL-17A should not be used as a surrogate of vitreous fluid IL-17A as a biomarker for monitoring progression of DR.

## Supporting information

S1 Fig Association of IL-17A level with other cytokine levels in aqueous humor of patients with proliferative diabetic retinopathy. Correlation of aqueous IL-17A level vs. aqueous levels of other cytokines was analyzed by Spearman’s correlation coefficient test. (A) IL-6: y = 2299–19.0x, R^2^ = 0.003, *P* = 0.778; (B) IL-10: y = 1.37 + 0.25x, R^2^ = 0.133, *P* = 0.044; (C) IL-17F: y = 33.9–0.09x, R^2^ = 0.0002, *P* = 0.9347; (D) IL-22: y = 0.79 + 0.27x, R^2^ = 0.147, *P* = 0.033; (E) IL-25: y = 1.86–0.03x, R^2^ = 0.007, *P* = 0.653; (F) IL-31: y = 8.97 + 0.09x, R^2^ = 0.002, *P* = 0.792; (G) IFN-γ: y = 11.2–0.03x, R^2^ = 0.0001, *P* = 0.953; (H) sCD40L: y = 14.6–0.12x, R^2^ = 0.002, *P* = 0.827; and (I) TNFα: y = 0.76 + 0.14x, R^2^ = 0.262, *P* = 0.003.
(TIF)
